# Cellular and Molecular Mechanisms of Anterior Chamber-Associated Immune Deviation (ACAID): What We Have Learned from Knockout Mice

**DOI:** 10.3389/fimmu.2017.01686

**Published:** 2017-11-30

**Authors:** Julie Vendomèle, Quentin Khebizi, Sylvain Fisson

**Affiliations:** ^1^INTEGRARE, Genethon, INSERM, Univ Evry, Université Paris-Saclay, Evry, France

**Keywords:** ACAID, knockout mice, cell deficiency, molecular deficiency, eye, ocular immune privilege

## Abstract

Anterior chamber-associated immune deviation (ACAID) is a well-known phenomenon that can occur after an antigen is introduced without any danger signal into the anterior chamber of a murine eye. It is reported to lead to an antigen-specific immune deviation throughout the body. Despite the relatively little evidence of this phenomenon in humans, it has been suggested as a potential prophylactic strategy in allograft rejections and in several autoimmune diseases. Cellular and molecular mechanisms of ACAID have been explored in different murine models mainly as proofs of concept, first by direct analyses of immune components in normal immunocompetent settings and by cell transfer experiments. Later, use of knockout (KO) mice has helped considerably to decipher ACAID mechanisms. However, several factors raise questions about the reliability and validity of studies using KO murine models. This mini-review summarizes results obtained with KO mice and discusses their advantages, their potential weaknesses, and their potential methods for further progress.

## Introduction

**Figure 1:**
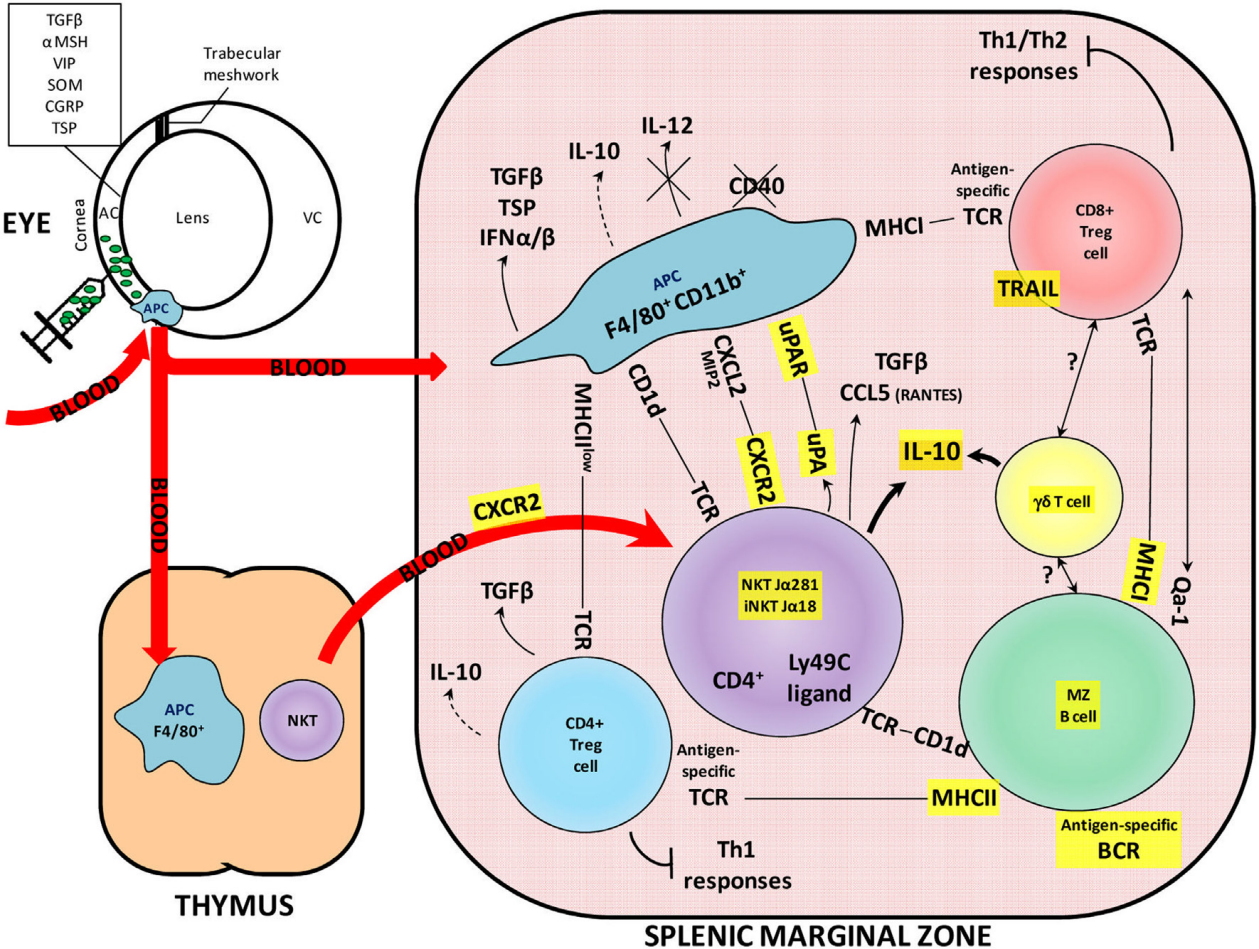
Overview of the contribution of KO mice to understanding the molecular and cellular interactions in the ACAID mechanism. The ACAID mechanism is an immunomodulatory phenomenon induced following an injection of an antigen into the anterior chamber of an eye. The antigen (depicted here as green particles) is taken up and processed by ocular APCs expressing the F4/80 and CD11b molecules. These APCs migrate *via* the blood to the thymus and the spleen and contribute to the generation of immunomodulatory cells. The importance of KO models in understanding ACAID is demonstrated by the use of these models in elucidating the roles of molecules highlighted in yellow. AC, anterior chamber; BCR, B-cell receptor; MZ, marginal zone; MHC, major histocompatibility complex; NKT, natural killer T cell; TCR, T-cell receptor; VC, vitreous chamber; KO, knockout; ACAID, anterior chamber-associated immune deviation; APCs, antigen-presenting cells.

For over a century, the eye has been considered as an immune-privileged site, for its local immune characteristics and its systemic modulation of antigen-specific immune responses. Both its local properties, such as the blood–retinal barrier and the secretion of immunosuppressive molecules (e.g., somatostatin, TGF-β), and some peripheral mechanisms, such as anterior chamber-associated immune deviation (ACAID), highlight the eye’s very specific and pro-tolerogenic status (1, 2). As demonstrated in different animal models, an antigen (e.g., ovalbumin) injected into the anterior chamber of the eye is taken up and processed by antigen-presenting cells (APCs) that migrate *via* the blood to the thymus and the spleen (Figure 1). The source of these APCs is still controversial: they may be recruited as monocytes from the blood after the injection (3) or, alternatively, could be resident APCs. In any case, these F4/80^+^ CD11b^+^ ocular APCs migrate to the thymus and induce the generation of NKT cells (NK1.1^+^ CD4^−^ CD8^−^), which, in turn, play a role in producing splenic suppressor cells. In the marginal zone (MZ) of the spleen, F4/80^+^ CD11b^+^ APCs emigrating from the eye also interact with various types of cells and molecules, helping to generate immunomodulatory cells, such as CD8^+^ or CD4^+^ regulatory T cells (Tregs), MZ regulatory B cells, γδ Tregs, iNKT, and NKT regulatory cells, which spread throughout the body and induce antigen-specific immune deviation (4). Among the immunologic hallmarks of ACAID are its inhibition, first, of the initial activation and differentiation of T cells into Th1 effector cells (mainly characterized by secretion of IFNγ, TNFα, and IL-2), and second, of the expression of Th1-mediated immunity, such as delayed-type hypersensitivity (DTH) and skin allograft rejection (5). Moreover, the demonstration that the induction of ACAID also mitigates Th2-mediated immune responses, such as allergic inflammatory lung diseases, leads to the conclusion that this mechanism is not only a cross-regulation of the Th1 immunity produced by a robust Th2 response (mainly characterized by secretion of IL-4, IL-10, and IL-13), but rather a complex immunoregulatory phenomenon that involves multiple organ systems and cell populations (6).

The ACAID mechanism exists in many mammalian species, including mice, rats, rabbits, and nonhuman primates (7–9). Although a direct and in-depth investigation of its presence in humans is difficult, the low rate of corneal allograft rejection and the sparse use of immune suppressors for these grafts compared with systemic transplants offer indirect proof of the pro-tolerogenic environment of the eye. Moreover, a Japanese study provided evidence of an ACAID-like mechanism in humans by testing DTH in both healthy controls and patients infected with ocular varicella-zoster virus (VZV). They showed that the controls displayed positive DTH in response to intradermal VZV, but that these responses were absent in 60% of the patients with ocular VZV. This finding thus revealed an immune deviation of the immune response toward VZV (10). Due to the difficulty of exploring ACAID mechanisms in humans, murine models have been widely used to study this immune process. The methodological approaches used have included splenectomy, thymectomy, monoclonal antibody (mAb) injections, cell transfers, and knockout (KO) mice; each of them has contributed to understanding ACAID and has confirmed or excluded the role of specific molecules and cell types. Splenectomy and thymectomy revealed the key role of these organs in the development of ACAID (11, 12). These approaches cannot, however, identify the cellular and molecular components involved in detail. Although mAbs display a limited half-life *in vivo* and induce only partial depletion due to the inaccessibility of targets in tissue or transient removal of the target from the cell surface (13), they have been informative in highlighting ACAID mechanisms (14). Another solution, albeit radical, for determining a molecule’s involvement in a physiological mechanism is definitively ensuring its absence: when certain molecules cannot be expressed in an animal, such as KO mice, specific cell types may not develop (15, 16). The difficulty in generating KO mice has long been an impediment in the use of this methodological approach, but this is no longer the case today, and many service companies offer KO mice relatively, rapidly, and inexpensively.

In this review, we examine the role that conventional (non-conditional) KO mice have played in deciphering and understanding the molecular and cellular interactions triggered in ACAID. We then discuss some important limitations of the KO mouse models used so far, as well as new alternatives to them.

## Use of KO Mice to Decipher ACAID Mechanisms

Knockout mice are generated by the replacement of an essential coding region in a genetic locus by a drug resistance marker (16), which prevents the expression of a molecule of interest; this in turn can lead to the absence of certain cell types. Table 1 summarizes the studies exploring ACAID mechanisms with KO mice according to whether or not the model of molecular deficiency is associated with a cellular deficiency.

**Table 1:** Molecular and/or cellular deficiencies associated with KO mice and impact on ACAID induction.

		KO molecule	Strain (haplotype)	ACAID is impaired	ACAID is inducible
With cell deficiency	B cells	IgM	B6.129S2-Igh^mtm1Cgn^/J or C57Bl/6-Igh-6^tm1Cgn^ (H2^b^)	(15)	
	
				(17)	
	T cells	FOXN1	*Nude (H2^d^)	(15)	
	CD4^+^ T cells	CD4	B6.129S2-Cd4^tm1Mak^ (H2^b^)	(18)	
	γδ T cells	TCRδ	C57Bl/6-TCRd^tm1 Mom^ (H2^b^)	(18)	
				(19)	
				(20)	
	NKT/iNKT cells	TCRα	Jα281 or Jα18 KO (H2^b^ or H2^d^)	(21)	
				(22)	
				(23)	
				(24)	
				(25)	

Without cell deficiency	Soluble molecules	IL-4	IL-4^tmlcgn129^ (H2^b^)		(26)
					(15)
					(27)
		IL-4 and IL-13	IL-4^−/−^/IL-13^−/−^ (H2^d^)		(24)
		IL-13	IL-13^−/−^ (H2^d^)		(24)
		IL-6	B6,129S2-IL6^tmKopf/J^ (H2^b^)		(28)
		IL-10	IL-10^tmlcgn129^ (H2^b^)	(15)	
				(27)	
		IFNγ	IFNγ^−/−^ (H2^b^)	(29)	
		Plasminogen	Plasminogen KO (H2^b^)	(25)	
		Substance P and neurokinin A	B6.Cg-Tac1^tm1Bbm^/J (H2^b^)		(28)
		TSP-1	TSP1 KO (H2^b^)	(30)	
		Urokinase-type plasminogen activator (uPA)	uPA KO (H2^b^)	(25)	

	Membrane molecules	CD1	CD1 KO (H-2^b^)	(31)	
		CXCR2	Cmkar2^tm1Mwm^ (H2^d^)	(32)	
		DR5	Dr5^−/−^ (H2^b^)	(33)	
		F4/80	B6.F4/80^−/−^ (H2^b^)	(34)	
		IFNγR	IFNγR^−/−^ (H2^b^)	(29)	
		TGFβRII	**CD4-dnTGFβRII (H2^b^)	(35)	
		TNFRI	TNFRI KO (H2^b^)		(36)
		TNFRII	TNFRII KO (H2^b^)	(36)	
		TNF-related apoptosis-inducing ligands (TRAIL)	Trail^−/−^ (H2^b^)	(33)	
		uPAR	uPAR KO (H2^b^)	(25)	
	
	Intracellular molecules	Cbl-b	Cbl-b KO (H2^b^)	(35)	
		STAT6	STAT6^−/−^ (C129.S2)		(24)

*When its gene is knocked, a protein cannot be expressed and a molecular deficiency occurs. In some cases, the lack of a specific protein induces a cell deficiency. Studies of several independent deficiencies have demonstrated the critical nature of the corresponding molecules in anterior chamber-associated immune deviation (ACAID) induction. The table classifies papers studying the ACAID mechanism with knockout (KO) mice according to the phenotypic impact of the KO (with or without cell deficiency). Depending on the molecule deficiency, these papers reported ACAID induction either continued (stated as “ACAID is inducible”) or was inhibited (“ACAID is impaired”)*.

*Nude mice are not strictly considered KO mice, but are intentionally included because their mutation in the Foxn1 gene induces an absence of mature T cells.

**CD4-TGFβRII mice are not strictly considered KO mice because they overexpress a dominant negative form of TGFβRII, that is, a nonfunctional receptor binding TGFβ with high affinity.

*H2^b^ and H2^d^ are the haplotypes of mouse strains used in the referenced studies*.

The use of mice that abolishes cellular compartment has helped researchers to understand both the innate and adaptive immunity involved in several steps of ACAID. Specifically, a deficiency in cells of either the adaptive system (i.e., B or T cells, especially CD4^+^ or γδ T cells) or the innate system (e.g., NKT cells) systematically impairs ACAID. T cells are of major interest in ACAID. In the late 1990s, D’Orazio and Niederkorn showed that ACAID induction was impaired in nude mice, as expected. Later on, experiments with more specific KO mice demonstrated that CD4^+^ T cells, γδ T cells, and NKT cells are all required to induce ACAID. No studies, however, appear to have taken place with CD8^+^ KO mice, even though these cells are essential for ACAID (29, 37). Skelsey and colleagues showed in 2003 that CD4^+^ T cells are required for the non-contact-dependent generation of CD8^+^ Tregs with CD4^+^ KO mice. Skelsey et al. used γδ T cell KO mice to show in 2001 that γδ T cells are required for the generation of regulatory cells in ACAID. In 2002, Xu et al. confirmed these results and reported that they are also essential for the inhibition of cytotoxic immune responses. More recently, in 2006, Ashour et al. showed that the tolerogenic characteristics of these γδ T cells are due to their secretion of IL-10, which induces the generation of Tregs.

Several studies have focused on the role of NKT cells in ACAID. In 2001, Sonoda et al. showed that IL-10 secreted by NKT cells during ACAID is necessary for the induction of specific Tregs. The following year, they reported that the CD1d molecule, which must interact with NKT cells to induce tolerance, is expressed by APCs from the eye and B cells from the MZ of the spleen. In 2003, Nakamura et al. showed that NKT cells involved in ACAID express the CD4 marker and are required for the generation of regulatory CD8^+^ T cells. Two years later, the same group reported that ACAID induction does not require that NKT cells secrete IL-4 and IL-13. Finally, in 2007, Sonoda et al. demonstrated that NKT cells secrete urokinase-type plasminogen activator (uPA), which is required for ACAID induction.

B cells also play a crucial role in ACAID, especially in the splenic phase. In 1998, D’Orazio and Niederkorn reported that ACAID could not be induced in B-cell deficient mice, but that the reconstitution of this compartment with wild-type B cells restored ACAID. Interestingly, ACAID APCs co-cultured with splenocytes from B-cell KO mice did not induce efferent suppressors of DTH. Further clarification of the role of B cells from the MZ came in 2002 from Sonoda et al. who identified these cells as presenters of the CD1d molecule to NKT cells. Finally, Ashour and Niederkorn reported in 2006 that B cells must simultaneously express major histocompatibility complex (MHC) class I and II molecules to generate Treg cells during ACAID. Thus, B cells, especially those from the MZ, appear essential for ACAID induction. Overall, these findings about the cellular mechanisms of ACAID using KO mice reveal a very complex and intricate process in which all cell partners seem to play crucial and non-redundant roles.

Several other models of KO mice produce molecular deficiencies without a cellular deficiency (Table 1); these can affect cell communication, T-cell polarization, inflammation, immune regulation, and cell migration. The molecules involved in direct cell–cell contact demonstrated to be essential to the ACAID mechanism are CD1 and F4/80. The CD1 allelic variants are non-polymorphic MHC class I-like proteins that present lipids and bacterial metabolites to T cells (38). Expression of the F4/80 molecule (also known as EMR1/EGF-like module-containing mucin-like hormone receptor-like 1, Ly71, Gpf480, TM7LN3, DD7A5-7, or EGF-TM7) on APCs is involved in the generation of efferent CD8^+^ T regulatory cells, as Lin et al. reported in 2005. Although it is not yet clear why this adhesion G protein-coupled receptor is essential for the function of APCs involved in ACAID, the F4/80 marker has already been used to identify tolerogenic APCs able to suppress autoimmune diseases, such as experimental autoimmune uveitis by induction of CD8^+^ Treg cells (39, 40).

Another category of molecules involved in cell communication that have been demonstrated to be essential to the ACAID mechanism comprises the secreted molecules and their receptors, including CXCR2, DR5, TNF-related apoptosis-inducing ligands (TRAIL), IFNγ, IFNγR, IL-4, IL-13, IL-6, IL-10, TGFβRII, TNFRI, TNFRII, uPA, uPAR, plasminogen, substance P, and neurokinin A (Table 1). The chemokine receptor CXCR2 and its ligands are mainly described as regulating neutrophil recruitment and neural repair mechanisms (41). Besides its involvement in neuroinflammatory brain pathologies, however, CXCR2 is crucial to the ACAID mechanism, since NKT cells are recruited by MIP-2, a ligand of CXCR2 (32). A lack of CXCR2 thus prevents the accumulation of NKT cells in the spleen and the subsequent generation of Tregs. The TRAIL, expressed by CD8^+^ Treg cells, and their receptor DR5 (Death receptor 5 or TRAIL receptor 2) also play an essential role in the peripheral suppression of cell-mediated immunity after antigen injection into the anterior chamber of the eye (33), through TRAIL T-cell homeostasis and differentiation: the so-called “helpless” CD8^+^ T cells, primed in the absence of CD4^+^ T-cell help, are unable to undergo a second round of clonal expansion upon restimulation with their cognate antigen because they are eliminated by activation-dependent killing *via* TRAIL. Moreover, Th1 cells can be killed by TRAIL, whereas Th2 cells are resistant to TRAIL-induced apoptosis (42). Since ACAID is mainly tested by Th1 responses (such as DTH), ACAID cannot be induced in TRAIL KO or DR5 KO mice. We might wonder how ACAID could be impaired in IFNγ or IFNγR deficient mice, since IFNγ is known to be down-regulated (29). As Falschlehner et al. (42) showed, however, IFNγ induces the expression of TRAIL on the surface of monocytes, dendritic cells (DCs), and natural killer (NK) cells. It is thus important to distinguish the positive local (i.e., ocular) involvement of IFNγ in ACAID from its systemic pro-inflammatory effect, which ACAID inhibits.

TNFα, another Th1 cytokine, also contributes to the generation of ACAID (43), by upregulating the Fas receptor and promoting Fas-induced apoptosis and the subsequent induction of ACAID. Although expression of TNFRII on corneal cells confers protection against immune rejection of corneal allografts, this mechanism is independent of ACAID (36). IL-4 and IL-13 KO mice have helped to demonstrate that these Th2-related molecules are not indispensable, since ACAID is inducible in their absence (15, 24, 26–28). It is nonetheless important to keep in mind that these studies have been conducted predominantly in C57Bl/6 mice, which have a pro-Th1 background. In contrast to IL-6, which is not essential for ACAID induction, IL-10 has been demonstrated to be indispensable (15, 24, 26–28). IL-10 can be produced by Th2 lymphocytes. This finding is nonetheless consistent with the previous demonstration that IL-4 and IL-13 are not necessary for ACAID induction, since IL-10 is an important soluble regulator produced by many different suppressive cells and should not be considered here as a Th2-associated molecule. IL-10 appears to play a central role in ACAID. Indeed, it is a master regulator of effector cells and helps to maintain APCs in a tolerogenic state, by directing the immune response away from a Th1 pathway and suppressing DTH (44). Interestingly, TGFβ predisposes ocular APCs to secrete IL-10 during antigen processing. This is consistent with the finding that TGFβ, a powerful inhibitory molecule (in the absence of IL-6), also seems to be required for ACAID, as demonstrated by the use of CD4-dnTGFβRII dominant-negative mice (35).

The plasminogen activation system, including plasminogen, uPA, and the urokinase receptor (also known as uPAR or CD87), is another important contributor to the ACAID mechanism, as Sonoda et al. demonstrated in 2007. It is not yet clear exactly how these molecules contribute to ACAID, but besides the production by cells from the retinal pigment epithelium (RPE) of plasminogen activators and expression of receptors for urokinase, which may allow local proteolysis and migration, the plasminogen activation system is involved in various other non-proteolytic processes, including cell migration, cell cycle regulation, and cell adhesion (45). Interestingly, RPE cells from TSP-1 null mice cannot either convert latent TGFβ into its active immune inhibitory form or inhibit T-cell activation (30). In contrast, the ability of mice KO for substance P and neurokinin to induce ACAID demonstrates that these molecules are not required for ACAID. More precisely, the secretion of these molecules is induced in the context of a retinal laser burn which in turn impairs ACAID (28).

The intracellular signaling Cbl-b molecule comprises the final category of molecules demonstrated to be essential to the mechanism of ACAID. Cbl-b is known to be a negative regulator of T-cell activation, controlling the extent of this activation during antigen presentation (46). Cone et al. demonstrated in 2009 that DTH reactions are not inhibited (that is, ACAID is not induced) in Cbl-b deficient mice; this is thought to be due to an abnormality in Cbl-b^−/−^ T cells in the phosphorylation of Smad2.

## Reliability and Validity of Studies Using KO Murine Models

The studies of ACAID with KO mice have predominantly used mice with a C57Bl/6 (H-2^b^) background. This mouse background is well-known for its pro-Th1 profile, and is often contrasted with BALB/c mice, which are considered to be pro-Th2 (47). Interestingly, BALB/c is the predominant animal model in the general experimental field of ACAID, but very few KO mice exist in this background, and they have not been used to test KO effects on ACAID. Moreover, bovine serum albumin (BSA) and ovalbumin (OVA) are frequently used as antigen models in experiments with ACAID, and BSA has not yet been used in KO mouse models. These points might bias the analysis of the results obtained with these KO mouse models that have been used. Finally, the most common outcome measure or endpoint used to assess ACAID models is footpad or ear measurement of the swelling caused by DTH. This variable provides evidence of the recruitment of inflammatory cells, especially macrophages, but gives no direct clue about either the involvement of regulatory cells or the Th1/Th2 profiles.

Despite the advantages of their ability to target several molecules and cell types and their easy availability, the physiological relevance of conventional KO mice can be contested, since the existence of compensatory mechanisms or potential physiological impacts of molecular and cellular deficiencies cannot be totally excluded. The KO murine models used to decipher ACAID mechanisms have been exclusively conventional. It would be interesting to explore the same questions with conditional KO mice to rule out the possibility of non-physiological conditions, with an effect that is not already present at birth and may be temporary. Although it has not yet been used for deciphering ACAID mechanisms, knock-in or KO mouse technology expressing the diphtheria toxin receptor (DTR) under the control of a cell-specific promoter (48) could be useful for conditional depletion of specific target cells *in vivo*. This kind of DTR mouse, however, presents problems related to the persistence of a non-negligible proportion of target cells (and their consequent weak expression of the receptor), as well as the inability of this strategy to target soluble molecules and the potential toxicity of *in vivo* use of diphtheria toxin.

Another alternative is the use of Cre/Lox system in conjunction with the selective and inducible expression of Cre-recombinase, by using a TetOn/TetOff system with doxycycline (49). This technology makes it possible to abrogate the expression of a given molecule in a specific cell type, at a precise time point. An interesting question to address with this technology is the central role of IL-10. More precisely, inhibiting IL-10 expression specifically in NKT or γδT cells (e.g., floxed IL-10 and cre expression under Jα281 or TCRd promoters, respectively) before, during, and after the induction of ACAID might pinpoint the cellular source of this crucial cytokine and provide precisions on the timing of its action. This approach may shed light on the potential non-redundant role of IL-10 producing NKT or γδT cells in ACAID induction. Furthermore, the sympathetic nervous system has been described as a major player in ACAID induction (50), but deeper studies could be performed to refine its precise role. The two major molecules involved in this pathway are acetylcholine and norepinephrine (51). To avoid possible lethal issues due to the KO of acetylcholine or norepinephrine in the whole organism, we suggest to KO their receptors specifically in lymphoid cells such as CD3^+^ cells, which actually express these receptors (52). This would help to refine the interactions between the immune system and the sympathetic nervous system in ACAID induction, especially highlighting the T-cell direct dependence to acetylcholine and norepinephrine in the production of splenic suppressor cells involved in ACAID.

Recent discovery of the clustered regularly interspaced short palindromic repeats (CRISPR/Cas9) system, a new powerful tool for genome editing, has opened up a wide range of applications, related in particular to site-specific knock-in and conditional KO (53). Despite some limitations due to the presence of potential off-target genomic sites, this approach should be used in the future to further decipher the mechanisms of ACAID in mice, but also in humans. For example, strategies to inhibit the immune tolerance of the eye in order to induce an antitumor immune response in ocular lymphomas could be designed by KO molecules such as EMR2 (F4/80 human counterpart) (54). Another conceivable track is that knocking out some genes, such as TAC1 encoding for substance P (28), might induce tolerance and thereby limit or control ocular inflammation.

## Conclusion

Animal models of ACAID have served as tools to identify key factors involved in the immune privilege of the eye. KO mice have helped considerably in decoding this mechanism, aiding in identifying essential cells and molecules. Despite the debatable physiological relevance of KO mice, techniques including cell staining and adoptive cell transfer experiments in physiological models have corroborated these findings. These KO mice have thus been fruitfully used to study ACAID and have set the essential foundations for the development of future immunomodulatory therapies.

## Author Contributions

JV, QK, SF: substantial contributions to the conception or design of the work. JV, QK, SF: drafting the work or revising it critically for important intellectual content. JV, QK, SF: final approval of the version to be published. JV, QK, SF: agreement to be accountable for all aspects of the work in ensuring that questions related to the accuracy or integrity of any part of the work are appropriately investigated and resolved.

## Conflict of Interest Statement

The authors declare that the research was conducted in the absence of any commercial or financial relationships that could be construed as a potential conflict of interest.

## Abbreviations

ACAID, anterior chamber-associated immune deviation; APCs, antigen-presenting cells; BSA, bovine serum albumin; DTH, delayed-type hypersensitivity; DTR, diphtheria toxin receptor; KO, knockout; MHC, major histocompatibility complex; mAbs, monoclonal antibodies; Tregs, regulatory T cells; RPE, retinal pigment epithelium; TRAIL, TNF-related apoptosis-inducing ligands; uPA, urokinase-type plasminogen activator.

## References

[1] Hori J Vega JL Masli S. Review of ocular immune privilege in the year 2010: modifying the immune privilege of the eye. Ocul Immunol Inflamm (2010) 18:325–33. 10.3109/09273948.2010.512696 20849282

[2] Niederkorn JY. The induction of anterior chamber-associated immune deviation. Chem Immunol Allergy (2007) 92:27–35. 10.1159/000099251 17264480

[3] Pais R Bhowmick S Chattopadhyay S Lemire Y Sharafieh R Yadav R An intracameral injection of antigen induces in situ chemokines and cytokines required for the generation of circulating immunoregulatory monocytes. PLoS One (2012) 7:e43182. 10.1371/journal.pone.0043182 22912822 PMC3422248

[4] Toscano-Tejeida D Ibarra A Phillips-Farfán BV Fuentes-Farías AL Meléndez-Herrera E. ACAID as a potential therapeutic approach to modulate inflammation in neurodegenerative diseases. Med Hypotheses (2016) 88:38–45. 10.1016/j.mehy.2016.01.006 26880635

[5] Stein-Streilein J Streilein JW. Anterior chamber associated immune deviation (ACAID): regulation, biological relevance, and implications for therapy. Int Rev Immunol (2002) 21:123–52. 10.1080/08830180212066 12424840

[6] Katagiri K Zhang-Hoover J Mo JS Stein-Streilein J Streilein JW. Using tolerance induced via the anterior chamber of the eye to inhibit Th2-dependent pulmonary pathology. J Immunol (2002) 1950(169):84–9. 10.4049/jimmunol.169.1.84 12077232

[7] Borrás T Gabelt BT Klintworth GK Peterson JC Kaufman PL. Non-invasive observation of repeated adenoviral GFP gene delivery to the anterior segment of the monkey eye in vivo. J Gene Med (2001) 3:437–49. 10.1002/jgm.210 11601757

[8] Eichhorn M Horneber M Streilein JW Lutjen-Drecoll E. Anterior chamber-associated immune deviation elicited via primate eyes. Invest Ophthalmol Vis Sci (1993) 34:2926–30. 8360025

[9] Li Z Peng G Li C. [The role of spleen in induction and maintenance of anterior chamber-associated immune deviation in different species of animals]. Yan Ke Xue Bao (1999) 15(221–224):237. 12579673

[10] Kezuka T Sakai J Usui N Streilein JW Usui M. Evidence for antigen-specific immune deviation in patients with acute retinal necrosis. Arch Ophthalmol (2001) 1960(119):1044–9. 10.1001/archopht.119.7.1044 11448326

[11] Streilein JW Niederkorn JY. Induction of anterior chamber-associated immune deviation requires an intact, functional spleen. J Exp Med (1981) 153:1058–67. 10.1084/jem.153.5.1058 6788883 PMC2186172

[12] Wang Y Goldschneider I Foss D Wu DY O’Rourke J Cone RE. Direct thymic involvement in anterior chamber-associated immune deviation: evidence for a nondeletional mechanism of centrally induced tolerance to extrathymic antigens in adult mice. J Immunol (1997) 1950(158):2150–5. 9036960

[13] Vaughan AT Cragg MS Beers SA. Antibody modulation: limiting the efficacy of therapeutic antibodies. Pharmacol Res (2015) 99:269–75. 10.1016/j.phrs.2015.07.003 26188150

[14] Skelsey ME Mellon J Niederkorn JY. Gamma delta T cells are needed for ocular immune privilege and corneal graft survival. J Immunol (2001) 1950(166):4327–33. 10.4049/jimmunol.166.7.4327 11254685

[15] D’Orazio TJ Niederkorn JY Splenic B cells are required for tolerogenic antigen presentation in the induction of anterior chamber-associated immune deviation (ACAID). Immunology (1998) 95:47–55. 10.1046/j.1365-2567.1998.00581.x 9767456 PMC1364375

[16] Iredale JP Demystified … gene knockouts. Mol Pathol MP (1999) 52:111–6. 10.1136/mp.52.3.111 10621830 PMC395683

[17] Ashour HM Niederkorn JY Peripheral tolerance via the anterior chamber of the eye: role of B cells in MHC class I and II antigen presentation. J Immunol (2006) 1950(176):5950–7. 10.4049/jimmunol.176.10.5950 16670303

[18] Skelsey ME Mayhew E Niederkorn JY. CD25+, interleukin-10-producing CD4+ T cells are required for suppressor cell production and immune privilege in the anterior chamber of the eye. Immunology (2003) 110:18–29. 10.1046/j.1365-2567.2003.01676.x 12941137 PMC1783020

[19] Xu Y Kapp JA. Gammadelta T cells are critical for the induction of anterior chamber-associated immune deviation. Invest Ophthalmol Vis Sci (2001) 104:142–8. 10.1046/j.0019-2805.2001.01285.x 11683953 PMC1783294

[20] Ashour HM Niederkorn JY Gammadelta T cells promote anterior chamber-associated immune deviation and immune privilege through their production of IL-10. J Immunol (2006) 1950(177):8331–7. 10.4049/jimmunol.177.12.8331 17142729

[21] Sonoda KH Faunce DE Taniguchi M Exley M Balk S Stein-Streilein J. NK T cell-derived IL-10 is essential for the differentiation of antigen-specific T regulatory cells in systemic tolerance. J Immunol (2001) 1950(166):42–50. 10.4049/jimmunol.166.1.42 11123275

[22] Sonoda KH Taniguchi M Stein-Streilein J. Long-term survival of corneal allografts is dependent on intact CD1d-reactive NKT cells. J Immunol (2002) 1950(168):2028–34. 10.4049/jimmunol.168.4.2028 11823540

[23] Nakamura T Sonoda K-H Faunce DE Gumperz J Yamamura T Miyake S CD4+ NKT cells, but not conventional CD4+ T cells, are required to generate efferent CD8+ T regulatory cells following antigen inoculation in an immune-privileged site. J Immunol (2003) 1950(171):1266–71. 10.4049/jimmunol.171.3.1266 12874214

[24] Nakamura T Terajewicz A Stein-Streilein J. Mechanisms of peripheral tolerance following intracameral inoculation are independent of IL-13 or STAT6. J Immunol (2005) 1950(175):2643–6. 10.4049/jimmunol.175.4.2643 16081840

[25] Sonoda KH Nakamura T Young HA Hart D Carmeliet P Stein-Streilein J. NKT cell-derived urokinase-type plasminogen activator promotes peripheral tolerance associated with eye. J Immunol (2007) 1950(179):2215–22. 10.4049/jimmunol.179.4.2215 17675481

[26] Kosiewicz MM Alard P Streilein JW. Alterations in cytokine production following intraocular injection of soluble protein antigen: impairment in IFN-gamma and induction of TGF-beta and IL-4 production. J Immunol (1998) 1950(161):5382–90. 9820512

[27] Gao Y Herndon JM Zhang H Griffith TS Ferguson TA. Antiinflammatory effects of CD95 ligand (FasL)-induced apoptosis. J Exp Med (1998) 188:887–96. 10.1084/jem.188.5.887 9730890 PMC2213381

[28] Lucas K Karamichos D Mathew R Zieske JD Stein-Streilein J. Retinal laser burn-induced neuropathy leads to substance P-dependent loss of ocular immune privilege. J Immunol (2012) 1950(189):1237–42. 10.4049/jimmunol.1103264 22745377 PMC3401345

[29] Paunicka K Chen PW Niederkorn JY. Role of IFN-γ in the establishment of anterior chamber-associated immune deviation (ACAID)-induced CD8+ T regulatory cells. J Leukoc Biol (2012) 91:475–83. 10.1189/jlb.0311173 22180630 PMC3289396

[30] Zamiri P Masli S Kitaichi N Taylor AW Streilein JW. Thrombospondin plays a vital role in the immune privilege of the eye. Invest Ophthalmol Vis Sci (2005) 46:908–19. 10.1167/iovs.04-0362 15728547

[31] Sonoda KH Exley M Snapper S Balk SP Stein-Streilein J. CD1-reactive natural killer T cells are required for development of systemic tolerance through an immune-privileged site. J Exp Med (1999) 190:1215–26. 10.1084/jem.190.9.1215 10544194 PMC2195676

[32] Faunce DE Sonoda KH Stein-Streilein J. MIP-2 recruits NKT cells to the spleen during tolerance induction. J Immunol (2001) 1950(166):313–21. 10.4049/jimmunol.166.1.313 11123307

[33] Griffith TS Brincks EL Gurung P Kucaba TA Ferguson TA Systemic immunological tolerance to ocular antigens is mediated by TRAIL-expressing CD8+ T cells. J Immunol (2011) 1950(186):791–8. 10.4049/jimmunol.1002678 PMC3075597 21169546

[34] Lin H-H Faunce DE Stacey M Terajewicz A Nakamura T Zhang-Hoover J The macrophage F4/80 receptor is required for the induction of antigen-specific efferent regulatory T cells in peripheral tolerance. J Exp Med (2005) 201:1615–25. 10.1084/jem.20042307 15883173 PMC2212925

[35] Cone RE Chattopadhyay S Sharafieh R Lemire Y O’Rourke J Flavell RA T cell sensitivity to TGF-beta is required for the effector function but not the generation of splenic CD8+ regulatory T cells induced via the injection of antigen into the anterior chamber. Int Immunol (2009) 21:567–74. 10.1093/intimm/dxp023 19325036 PMC2675031

[36] Niederkorn JY Mayhew E Mellon J Hegde S. Role of tumor necrosis factor receptor expression in anterior chamber-associated immune deviation (ACAID) and corneal allograft survival. Invest Ophthalmol Vis Sci (2004) 45:2674–81. 10.1167/iovs.04-0144 15277491

[37] Jiang L He H Yang P Lin X Zhou H Huang X Splenic CD8+ T cells secrete TGF-beta1 to exert suppression in mice with anterior chamber-associated immune deviation. Graefes Arch Clin Exp Ophthalmol (2009) 247:87–92. 10.1007/s00417-008-0947-8 18797912

[38] Pedersen IR Lymphocytic choriomeningitis virus RNAs. Nature New Biol (1971) 234:112–4. 10.1038/newbio234112a0 5002277

[39] Araç D Boucard AA Bolliger MF Nguyen J Soltis SM Südhof TC A novel evolutionarily conserved domain of cell-adhesion GPCRs mediates autoproteolysis. EMBO J (2012) 31:1364–78. 10.1038/emboj.2012.26 22333914 PMC3321182

[40] Hsu S-M Mathew R Taylor AW Stein-Streilein J Ex-vivo tolerogenic F4/80^+^ antigen-presenting cells (APC) induce efferent CD8^+^ regulatory T cell-dependent suppression of experimental autoimmune uveitis. Clin Exp Immunol (2014) 176:37–48. 10.1111/cei.12243 24266626 PMC3958152

[41] Scott H Sollid LM Brandtzaeg P Expression of MHC class II determinants by jejunal epithelium in coeliac disease. J Pediatr Gastroenterol Nutr (1988) 7:145–6. 10.1097/00005176-198801000-00027 3335977

[42] Falschlehner C Schaefer U Walczak H Following TRAIL’s path in the immune system. Immunology (2009) 127:145–54. 10.1111/j.1365-2567.2009.03058.x 19476510 PMC2691779

[43] Elzey BD Griffith TS Herndon JM Barreiro R Tschopp J Ferguson TA. Regulation of Fas ligand-induced apoptosis by TNF. J Immunol (2001) 1950(167):3049–56. 10.4049/jimmunol.167.6.3049 11544288

[44] D’Orazio TJ Niederkorn JY A novel role for TGF-beta and IL-10 in the induction of immune privilege. J Immunol (1998) 1950(160):2089–98. 9498745

[45] Elner SG. Human retinal pigment epithelial lysis of extracellular matrix: functional urokinase plasminogen activator receptor, collagenase, and elastase. Trans Am Ophthalmol Soc (2002) 100:273–99. 12545698 PMC1358967

[46] Naramura M Jang I-K Kole H Huang F Haines D Gu H. c-Cbl and Cbl-b regulate T cell responsiveness by promoting ligand-induced TCR down-modulation. Nat Immunol (2002) 3:1192–9. 10.1038/ni855 12415267

[47] Kelso A Troutt AB Maraskovsky E Gough NM Morris L Pech MH Heterogeneity in lymphokine profiles of CD4+ and CD8+ T cells and clones activated in vivo and in vitro. Immunol Rev (1991) 123:85–114. 10.1111/j.1600-065X.1991.tb00607.x 1684785

[48] Goren I Allmann N Yogev N Schürmann C Linke A Holdener M A transgenic mouse model of inducible macrophage depletion: effects of diphtheria toxin-driven lysozyme M-specific cell lineage ablation on wound inflammatory, angiogenic, and contractive processes. Am J Pathol (2009) 175:132–47. 10.2353/ajpath.2009.081002 19528348 PMC2708801

[49] Sauer B. Inducible gene targeting in mice using the Cre/lox system. Methods (1998) 14:381–92. 10.1006/meth.1998.0593 9608509

[50] Li X Taylor S Zegarelli B Shen S O’Rourke J Cone RE. The induction of splenic suppressor T cells through an immune-privileged site requires an intact sympathetic nervous system. J Neuroimmunol (2004) 153:40–9. 10.1016/j.jneuroim.2004.04.008 15265662

[51] McCorry LK. Physiology of the autonomic nervous system. Am J Pharm Educ (2007) 71(4):78. 10.5688/aj710478 17786266 PMC1959222

[52] Fujii T Mashimo M Moriwaki Y Misawa H Ono S Horiguchi K Physiological functions of the cholinergic system in immune cells. J Pharmacol Sci (2017) 134:1–21. 10.1016/j.jphs.2017.05.002 28552584

[53] D’Agostino Y D’Aniello S. Molecular basis, applications and challenges of CRISPR/Cas9: a continuously evolving tool for genome editing. Brief Funct Genomics (2017) 16(4):211–6. 10.1093/bfgp/elw038 28057617

[54] Song H EGF module-containing mucin-like hormone receptor 2 and its role in human immune privilege, master thesis, Boston University, Massachusetts (2014).

